# EEG frequency tagging dissociates between neural processing of motion synchrony and human quality of multiple point-light dancers

**DOI:** 10.1038/srep44012

**Published:** 2017-03-08

**Authors:** Nihan Alp, Andrey R. Nikolaev, Johan Wagemans, Naoki Kogo

**Affiliations:** 1 Laboratory of Experimental Psychology, Brain & Cognition Research Unit, University of Leuven (KU Leuven), Leuven, Belgium; 2 Laboratory for Perceptual Dynamics, Brain & Cognition Research Unit, University of Leuven (KU Leuven), Leuven, Belgium

## Abstract

Do we perceive a group of dancers moving in synchrony differently from a group of drones flying in-sync? The brain has dedicated networks for perception of coherent motion and interacting human bodies. However, it is unclear to what extent the underlying neural mechanisms overlap. Here we delineate these mechanisms by independently manipulating the degree of motion synchrony and the humanoid quality of multiple point-light displays (PLDs). Four PLDs moving within a group were changing contrast in cycles of fixed frequencies, which permits the identification of the neural processes that are tagged by these frequencies. In the frequency spectrum of the steady-state EEG we found two emergent frequency components, which signified distinct levels of interactions between PLDs. The first component was associated with motion synchrony, the second with the human quality of the moving items. These findings indicate that visual processing of synchronously moving dancers involves two distinct neural mechanisms: one for the perception of a group of items moving in synchrony and one for the perception of a group of moving items with human quality. We propose that these mechanisms underlie high-level perception of social interactions.

Synchronous motion is frequently found in the animal kingdom: flocks of birds fly together in harmony, schools of fish swim in perfect unison, orcas hunt by navigating their motion in perfect synchrony. Humans are no exception. Synchrony might have been important in our evolution as a social species because it facilitates psychological unification in a cooperative society^1^. Moreover, synchronous motion is used to create choreography, which we often find appealing. Who was not impressed by the perfectly in-sync performance in the opening ceremony of the Summer Olympics of 2008 in Beijing? Synchronous motion is also not particular to animate creatures. It is also applied to the movements of man-made things, such as multiple swings, flying drones, fireworks exploding together (see examples here: http://gestaltrevision.be/s/SynMotion). How does the brain process these synchronized motions? To what extent is the synchrony of human motions special as opposed to inanimate synchronous motions?.

Until now, studies of neural mechanisms underlying motion perception focused mostly on low-level coherent motion, i.e., common fate^2^,^3^. Another group of studies considered higher-level visual processing of biological motion^4^,^5^,^6^,^7^. This line of studies was initiated in 1973 by Johansson, who has shown that only few point-lights attached to the joints of a human carrying out a specific action (e.g., walking, running, jumping) are sufficient for us to perceive a specific biological motion^8^. Such point-light displays (PLDs) are capable to carry specific information about the biological nature of the motion without other cues, such as familiarity, shape and color^8^. Therefore, PLDs have been used frequently to investigate perception of motion of single human figures^4^,^5^,^6^. Later, multiple PLDs were used to study high-level perception of motion in a social context, including meaningful interactions between agents involved in reciprocal actions or reacting to each other^9^,^10^,^11^. These studies revealed that detection of interacting PLDs (e.g., dancing together) is unaffected by spatial scrambling of their parts^5^ and that motion information alone is sufficient to detect the emotional state of the dancers^11^.

Thus, previous research of visual processing of synchronous motion has considered either low-level detection of simple coherent motion or high-level perception of reciprocal human actions. However, we believe that the video examples given above constitute a special case of group motion, which, to the best of our knowledge, was never studied before. We propose that this type of motion is processed on an intermediate level of the visual hierarchy and may involve two components: motion synchrony and human quality (i.e., looks and moves like a human) of a group. Therefore, in this study we ask whether inter-item motion synchrony is processed in the same way for a group of humans and non-humans, and whether a group of moving humans is processed in the same way for synchronous and asynchronous motions.

We address these questions by using the *frequency-tagging technique*^12^,^13^, which involves recording of brain responses to periodic stimulation with EEG. Specifically, in frequency-tagging, rapid contrast modulation with different frequencies is applied to different parts of visual stimuli. Under such periodic visual stimulation, the brain produces periodic responses at the frequencies of stimulation (fundamental frequencies) and their harmonics (for a review, see ref. 14). Most importantly, the brain may also generate a response at the frequency which is a combination of the given frequencies, e.g., f_1_ + f_2_, 2f_1_ + f_2_. These emergent responses, so-called *intermodulation* (IM) components, occur as a result of non-linear interactions between fundamental frequencies^15^,^16^. With IM components, the neural responses are detected as discrete signals, which is advantageous in comparison to detecting changes in amplitudes of given (i.e., fundamental) frequencies, because IM components depend on both the individual tagged stimulus elements and their interactions. In our study, we applied frequency-tagging to a group of four PLDs^8^ in a 2 × 2 experimental design, which combines the motion type (synchronous vs. asynchronous) and the human quality of the configurations (human vs. non-human) of the groups of PLDs. To preview our results, we detected two distinct IM components: one of them was associated with the motion synchrony and another one was associated with the human quality of the group of PLDs. These findings indicate that two independent neural mechanisms are involved in the perception of synchronous human motion.

## Results

**Figure 1:**
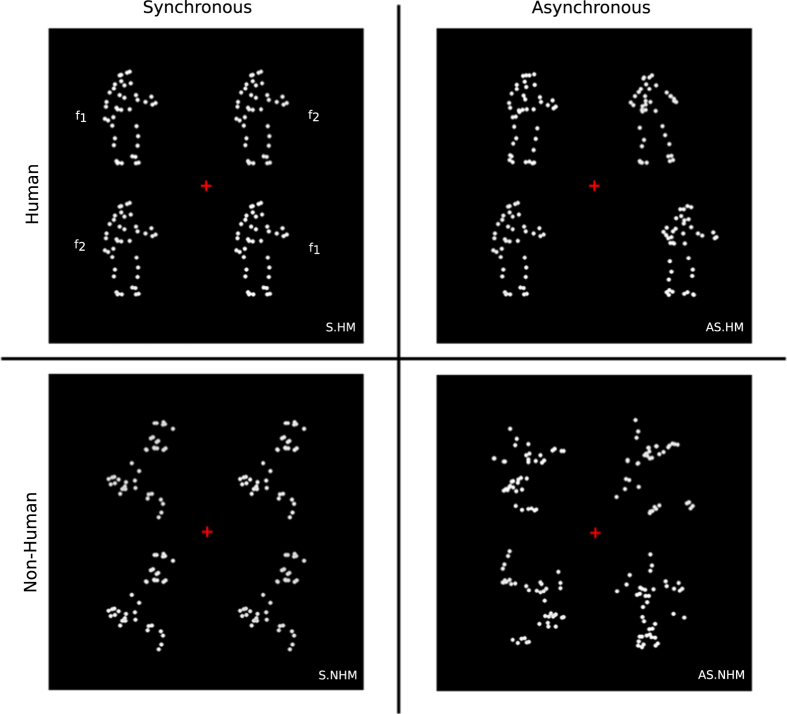
Four conditions of 2 × 2 experimental design. In the S.HM condition, the motions of four PLDs were perfectly in-sync. In the AS.HM condition, the synchrony between the dancers was destroyed. In the S.NHM condition, the body parts of all four PLDs were shuffled in the same way and the shuffled PLDs moved in synchrony. In the AS.NHM condition, the body parts of the PLDs were shuffled differently and the individual body parts started their motion from randomly selected frames. Point lights of a single PLD changed their contrast between white and dark-grey at a particular frequency. The two diagonal PLD pairs changed their contrast at different frequencies (f_1_ and f_2_) for all of the conditions.

We created a 10-s movie by juxtaposing four movies of PLDs^8^ into a single display and made four experimental conditions (Fig. 1, see video at http://gestaltrevision.be/s/BioMotion2x2Design). To this end, we concatenated a motion sequence of a PLDs^8^ with its reversed sequence. This concatenation created closed-loop motions, which enabled starting a movie from any frame, while preserving overall smooth motion. The combination of the forward and reversed sequences was essential because it allowed us to construct a long movie without repeating a motion cycle (see Stimuli section for more information). Moreover, by using the closed-loop motion, the PLDs can start to move from any frame without disturbing the motion smoothness. Later, we juxtaposed four of these movies into a single display and created four conditions by manipulating the human configuration and motion type of the PLDs as follows. In two conditions, human configurations were kept intact and while PLDs were dancing in unison in the synchronous human motion (S.HM) condition (Fig. 1, left-top panel), they were dancing independently in asynchronous human motion (AS.HM) condition (Fig. 1, right-top panel). Since it was shown that inverted motion disrupts animacy^17^ in two other conditions, the human configuration of the PLDs was destroyed by shuffling the body parts and turning the PLDs upside down. They were moving synchronously in the synchronous non-human motion (S.NHM) condition (Fig. 1, left-bottom panel), while they were moving asynchronously in the asynchronous non-human motion (AS.NHM) condition (Fig. 1, right-bottom panel). We applied rapid contrast modulation to all point-lights of one diagonal PLD pair with one frequency (f_1_) and all point-lights of another diagonal PLD pair with another frequency (f_2_).

38 participants were asked to look at the central fixation cross and to perform an orthogonal task of detecting a brief color change of the frame surrounding a display with four PLDs while EEG was recorded. Among the 38 participants (20 females, age range: 18–37), 24 participated in the first series and 14 participated in the second series of the experiment. They showed high performance for the behavioral task (99% correct).

**Figure 2:**
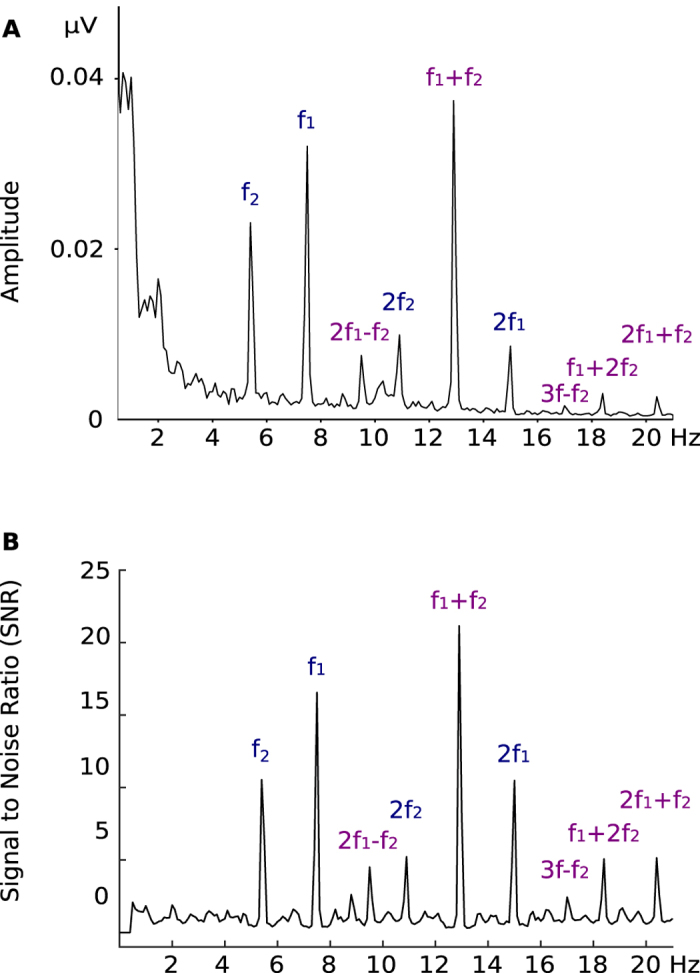
Amplitude spectrum (**A**) and signal-to-noise ratio (**B**) from 0 to 20 Hz for the frequency set f_1_ = 7.50 and f_2_ = 5.45 Hz, averaged across participants in the S.HM condition. The labels for fundamental (i.e., f_1_, f_2_) and harmonic frequencies (e.g., 2f_2_, 2f_1_) are in blue, while the labels for the IM components (e.g., f_1_ + f_2,_ 2f_1_ + f_2_) are in purple.

To test whether the expected effects depend on the choice of the tagging frequencies and to increase the reliability of the results, we applied two sets of frequencies to two groups of participants. We segmented the EEG recordings into trials corresponding to 10-s presentations of movies. After averaging EEG trials for each condition and each participant separately, we computed the amplitude spectrum with the fast Fourier transform (Fig. 2A).

**Figure 3:**
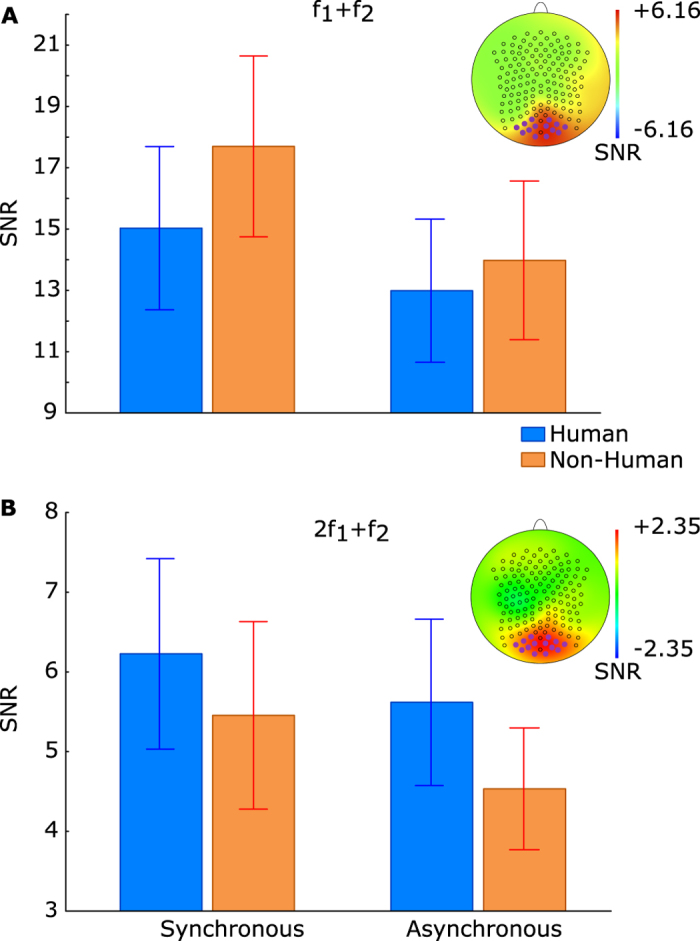
Effects of motion type and human configuration of a group of PLDs on SNR at the intermodulation components. (**A**) the effect of motion synchrony on SNR at the second-order IM component (f_1_ + f_2_): the SNR is significantly higher for a group with synchronous motions than for a group with asynchronous motion. (**B**) the effect of human configuration on SNR at the third-order IM component (2f_1_ + f_2_): the SNR is significantly higher for a group of human than non-human configuration. The columns indicate the SNR means and the error bars indicate the standard errors across participants. The maps show the differences of SNRs between the conditions and were computed as follows: (S.H + S.NH)-(AS.H + AS.NH) for motion type and (S.H + AS.H)-(S.NH + AS.NH) for human configuration. 14 purple circles on the maps indicate the occipital electrodes which were used in the analysis. The larger SNR scale for the second-order IM component (f_1_ + f_2_, the panel **A**) than for the third-order IM component (2f_1_ + f_2_, the panel B) reflects the trivial decrease of SNR with spectral frequency.

Next, we selected 14 occipital electrodes with a maximal amplitude at the fundamental frequencies (indicated by the purple circles on the maps in Fig. 3). To find the most prominent frequency components, we calculated Z-scores (per frequency set) of each frequency component. For each frequency set, the Z-score thresholding revealed seven common frequency types: two fundamentals, two harmonics and three IM components: one second-order (f_1_ + f_2_) and two third-order (2f_1_ + f_2_ and f_1_ + 2f_2_). We calculated a signal-to-noise ratio (SNR^18^,^19^) of each frequency type found in both datasets (Fig. 2B).

A repeated-measures ANOVA on the SNR values with the factors of motion type (synchronous vs. asynchronous) and human configuration (human vs. non-human) of the groups of PLDs showed no significant main effects for fundamental (f_1_ and f_2_) frequencies (Table 1). At f_2_ we found an interaction between motion type and human configuration (F(1, 37) = 6.43, p = 0.01, η^2^ = 0.15). At the level of harmonics, we did not observe any significant effects or interactions (see Table 1). However, among three IM components the effect of motion type was significant for the second-order IM component (f_1_ + f_2_): F(1, 37) = 16.78, p < 0.001, η^2^ = 0.31 (Fig. 3A) and the effect of human configuration was significant for one of two third-order IM components (2f_1_ + f_2_): F(1, 37) = 4.39, p = 0.04, η^2^ = 0.10 (Fig. 3B). The other third-order IM component (f_1_ + 2f_2_) showed the same pattern as 2f_1_ + f_2_, but the effect of human configuration did not reach significance: F(1, 37) = 3.11, p = 0.08, η^2^ = 0.07). The maps of the SNR values revealed that the effects of both factors were most prominent over the occipital areas (Fig. 3).

To check whether there is a similar pattern of results in each experimental series, we ran ANOVAs with the same design on the SNR values for each series separately (Table S1A,B). The effect of motion type, which was found for the second-order IM component (f_1_ + f_2_), was prominent in the first series, but there was no trend for this effect in the second series (Fig. S1A). The trends for the effect of human configuration, which was found for the third-order IM component (2f_1_ + f_2_), were observed in both series (Fig. S1B), although they did not reach significance (Table S1A,B). The results of these separate tests showed that in 3 separate cases the trends were in the same direction as the effects observed in the analysis of the merged series, and there were no trends in the opposite direction. Therefore, merging the two series did not qualitatively change the results and only increased the statistical power of the analysis.

To ensure that the results of the main analysis are not biased because of the different sample sizes in each series (24 and 14) we randomly selected 14 participants from the first series and repeated all analyses for 28 (14 + 14) participants. The results for IM components remained the same as in the main analysis (Table S2) with an effect of motion type for the second-order IM component (f_1_ + f_2_), an effect of human configuration for the third-order IM component (2f_1_ + f_2_), and a clear tendency for an effect of human configuration for the other third-order IM component (f_1_ + 2f_2_).

## Discussion

Applying the EEG frequency-tagging technique has allowed us to disentangle the neural processes underlying perception of synchronous biological motion. As mentioned before, the main advantage of this method is the possibility to reveal emerging neural responses (IM components) resulting from non-linear interactions in the brain^14^,^20^,^21^. The crucial feature of our experimental design is that IM components stem from interactions between the PLDs but not between the dots within a single PLD. In this way, IM components can only emerge as a result of long-range neural interactions between the signals coming from separate PLDs moving in a group. Thus, IM components necessarily signify perception of the PLD group as a whole. Since in our study, different frequencies were given to the two diagonal pairs of PLDs, the IM components must reflect global relationships between the two diagonal PLDs.

We found two distinct IM components, which correspond to the different types of information in displays with multiple moving items. The lower-order IM component (second-order IM: f1 + f2) is associated with a group of PLDs moving in synchrony and higher-order IM component (third-order IM: 2f1 + f2) is associated with a group of PLDs with human quality. The factors of motion synchrony and human quality are independent of the specific tagging frequencies, are most prominent over the occipital areas, and do not interact for either of the two IM components, providing no evidence for their dependence. In other words, we detected two distinct neural signals, one reflecting the motion synchrony no matter whether the items are human or non-human, and the other reflecting a group of moving humans no matter whether they move in synchrony or not.

Since the stimulation frequencies were applied to pairs of PLDs which moved synchronously or asynchronously within a pair, one can think that the effects of motion synchrony and human configuration can also be seen in the fundamentals or the harmonics. In general, we did not observe the effects on the fundamentals, except in the separate analysis of the second series, where f_1_ showed main effects of both motion synchrony and human configuration (Table S1B). The absence of the effects on the fundamentals is probably a consequence of lower sensitivity of the fundamentals than the IM components to interactions between PLDs^14^.

Whereas emergence of an IM component responsive to motion synchrony across PLDs is quite intuitive, emergence of an IM component responsive to human quality might be less obvious because human quality could be derived from the properties of a single PLD. Indeed, previous studies showed that even severe distortion of a human-like PLD does not eliminate its animacy^17^. However, in our design the IM components responsive to human quality result from the interaction of signals coming from multiple PLDs. Therefore, in our study “human quality” is an attribute of a group and not of an individual. To the best of our knowledge, such a distinction between perception of a *group* of humans vs. a *group* of non-humans has never been reported before.

Our findings indicate that perception of synchronously moving human bodies involves two distinct mechanisms: one for the processing of motion synchrony and one for the processing of a group of PLDs with human quality. The absence of interaction effects in our 2 × 2 design suggests that the synchronously moving human bodies in our displays do not trigger a higher-level, specialized mechanism for perception of social interactions. These interactions could be expected from previous studies which showed that even scrambled PLDs may contain information about reciprocal actions and reactions^9^,^10^. The key requirement for preserving perception of interactions after scrambling the PLD parts is congruency between the intrinsic joint motion and the extrinsic whole-body motion^10^. However, in our study, the scrambled PLDs were turned upside down. Inverting PLDs is known to disrupt the perception of animacy^17^ and therefore it is unlikely that even the synchronous non-human motion condition (S.NHM) gives rise to the perception of higher-level social interaction. Instead, our results suggest that perception of motion synchrony and human quality occurs at a processing level which is lower than the processing of social interaction. At such *intermediate level*, motion synchrony and human quality are processed independently, although it cannot be excluded that outcomes of their processing may later converge into a unified representation, where the social aspects are processed.

Within the intermediate level of the visual hierarchy, the order of an IM component may specify a distinct level of neural responses. As an IM component results from non-linear neural interactions^14^,^20^,^21^, the further the neural signal flows through the visual hierarchy, the more non-linear neural processes it involves. Specifically, at the early level of the hierarchy, the neural system may execute a simple non-linear operation. While the result of this operation is sent to the higher level, further non-linear operations are applied to the signals. This cascade of non-linear operations may result in emerging additional higher-order IMs as the signals reach to the higher level. For example, assume that non-linear operation is a square of the input *S*. The output, *S*^*2*^, is now sent to the next level and the same non-linear operation is applied to it which results in *S*^*4*^ as the output. With accumulating non-linear operations, the higher-order IMs become more and more prominent. Therefore, a higher-order IM component may reflect a response from a higher level of visual processing than a lower-order IM component. Applied to our findings, the lower-order IM component associated with perception of motion synchrony (the second-order IM: f1 + f2) suggests that within the intermediate level of the visual hierarchy it is processed earlier than the human quality of a group of PLDs (the third-order IM: 2f1 + f2). Note that another third-order IM (f1 + 2f2) also showed a tendency for the human quality effect but not for the motion synchrony effect. Thus, the perceptual mechanism of motion synchrony involves more basic processes than the perceptual mechanism for the human quality of a group of PLDs. This makes sense from an evolutionary point of view. Motion synchrony may be detected as basic information throughout evolution, whereas the motion of a group of humans may be detected as a more specific source of information which evolves later. Both perceptual mechanisms may then underlie further development of high-level processing of social interactions.

## Methods

### Participants

41 healthy adults having normal or corrected-to-normal vision participated in two series of the experiment. Experiments were identical except for the fundamental frequencies used for the frequency-tagging, as we explain next. One male and two females were discarded from the analysis due to technical issues during EEG recording and due to the low amplitude of the spectral components (see EEG Analysis). All participants, who signed the informed consent before the experiment were naive to the aim of the experiment. They were paid 8 euros per hour for participation. The ethical committee of the Faculty of Psychology and Educational Sciences of KU Leuven approved the experimental procedure and the experiment was conducted in accordance with the committee’s guidelines.

### Stimuli

The stimuli involved moving point-light displays (PLDs) and were constructed as follows. The movie with a stick dancer (“lindyHop2”) was selected from the Carnegie Mellon University Motion Capture Database (http://mocap.cs.cmu.edu/). The coordinates of the stick dancer in the original movie were converted to 41 point-lights of a PLD using the biological motion toolbox^22^ for MATLAB (MathWorks Inc., Natick, MA). We took the first 300 frames to make a 5-s movie. Next, the order of the same frames was reversed and the reversed frames were appended to the first 300 frames resulting in 600 frames of a 10-s movie. In the movie, a PLD dancer finished a complete cycle of a dancing motion and came back to the starting position.

A stimulus screen included four PLDs placed in the centers of four quadrants of a rectangle of 7.7° × 9.1° of visual angle. The size of a point light was 0.2° and the size of a PLD was 2.0° × 4.0° of visual angle at the viewing distance of 57 cm. The stimuli were presented on a black background at a LCD monitor (Dell E2010H, 17-inch size with resolution of 1600 × 900 and the refresh rate of 60 Hz) using a homemade program written in PsychoPy^23^. A stimulus screen was outlined with the blue or red contour and had a fixation cross in the middle.

In the S.HM condition we used four PLDs which all started their movements from the same frame and followed the same motion sequence. This stimulus was perceived as four human figures dancing in synchrony. In the AS.HM condition; the synchrony of motion between human figures was destroyed: each PLD started its motion from a different frame. For example, if a PLD started from the 10^th^ frame, it followed the entire sequence until the 600^th^ frame, after which it continued from the first to the 9^th^ frame. (Such motion looked smooth because of the closed-loop motion of the 600 frames.) In this way, the synchrony of dance in a group was disturbed but the human configuration of the PLDs was preserved. Two other conditions involved “parts-shuffled” PLDs. We decomposed the PLDs into nine clusters, each of which consisted of a few point-lights corresponding to the nine body parts: head, left arm, right arm, body, hips, left leg, right leg, left foot, and right foot. Next, we shuffled the Y-positions of the clusters and then turned the resulting PLDs upside down. By doing this, we eliminated the perception of a human-body configuration without changing the overall distance between the point-lights. In the S.NHM condition we used four parts-shuffled PLDs, i.e., the clusters of the four PLDs were shuffled in the same way and the motion started from the same frame. This stimulus was perceived as a synchronous motion of four identical objects. In the AS.NHM condition we disturbed both the human configuration and synchrony of motion by using four different parts-shuffled PLDs. To this end, we shuffled the Y-positions of the clusters differently for the individual PLDs. Furthermore, each cluster started the motion from a different frame, which was randomly selected. This stimulus was perceived as four objects moving without synchrony. In both parts-shuffled PLD conditions, PLDs were always the same across trials, and therefore spatial variability of our stimuli was equal across all conditions.

### EEG frequency-tagging

The contrast of the point-lights of a PLD was modulated sinusoidally between white and dark-grey (25% of greyscale). Two diagonal pairs of PLDs in a stimulus screen were tagged by two different frequencies. In the first series, the frequencies were f_1_ = 7.50 Hz and f_2_ = 5.45 Hz; in the second series, the frequencies were f_1_ = 4.00 Hz and f_2_ = 2.86 Hz. The diagonal arrangement of the frequency tagging equalized the possible hemispheric dominance for one of the frequencies.

The tagging frequencies were selected to meet the following five constraints. First, the frequency value had to be a product of a division of the monitor refresh rate by an integer, i.e., 60/11 = 5.45, 60/8 = 7.50 for the first series of the experiment and 60/21 = 2.86, 60/15 = 4.00 for the second series. Second, the meaningful frequencies (fundamentals, harmonics, and their combinations) should not coincide with each other (e.g., f_1_ + f_2_≠2f_1_ or f_1_ + f_2_≠2f_2_). Third, the meaningful frequencies had to be separated at least by 5 bins of the frequency spectrum (this was needed for computation of the Z-score and signal-to-noise ratio (SNR), as we will explain below). Fourth, the meaningful frequencies had to avoid the alpha frequency band of EEG (8–12 Hz), since SNR can be reduced if the frequencies appear within this band^14^. For this purpose, both the fundamental frequencies and the second-order sum intermodulation (IM) components (f_1_ + f_2_) were chosen outside the alpha band (i.e., 12 Hz < 5.45 + 7.50 = 12.95 Hz and 2.86 + 4.00 = 6.86 Hz < 8 Hz). Fifth, because the low frequencies produce more robust brain responses and can penetrate the higher level of visual processing more easily than the high frequencies^14^,^21^,^24^ the frequencies in the low range were chosen (2–8 Hz).

### Procedure

Participants were seated in a dimly lighted, sound-proofed and electrically shielded chamber. The moving stimuli were presented for 10-s with an inter-trial interval of 3 s. During a trial, the contour that outlined the stimulus screen changed its color from blue to red for 300 ms at a random time between zero to four times per trial. The participants’ task was to respond to the color change by pressing the “space” key of a keyboard. We instructed participants to look at the fixation cross while also spreading their attention over the entire screen in order to notice the type of motion – after the experiment the participants were asked to describe the motion. All participants reported that they perceived all types of motion, which were humans dancing together in the S.HM condition, or on their own in the AS.HM condition, some “monsters” moving in the same way in the S.NHM condition, and four objects or figures, each moving in different ways in the AS.NHM condition.

Each condition was repeated 10 times in a random-order in each block. This was repeated four times, resulting in 40 presentations of each condition. Two to five minute breaks were given between the blocks. The experiment lasted an hour and a half including preparation and breaks.

### EEG recording

EEG was recorded with a 256-channel Electrical Geodesics System (EGI, Eugene, Oregon, USA) using Ag/AgCl electrodes incorporated in a HydroCel Geodesic Sensor Net. The electrode montage included channels for recording vertical and horizontal electrooculogram (EOG). Impedance was kept below 50 kΩ. The vertex electrode Cz was used as a reference. The EEG was sampled at 250 Hz. All channels were preprocessed on-line using 0.1 Hz high-pass and 100 Hz low-pass filters.

### EEG Analysis

EEG analysis was done with BrainVision Analyzer (Brain Products GmbH, Gilching, Germany) and MATLAB. To remove the slow drift and high-frequency noise, which may affect artifact detection, we filtered the EEG using a Butterworth band-pass filter with low cutoff frequency at 0.53 Hz and high cutoff frequency at 45 Hz (and the notch filter at 50 Hz). We segmented the EEG into 10-s trials starting from the motion onset. We excluded trials in the following situations: the absolute voltage difference exceeded 50 μV between two neighboring sampling points; the amplitude was outside ± 100 μV; or the amplitude was lower than 0.5 μV during more than 100 ms, in any channel. On average, 5% of trials per participant were rejected because of artifacts. We averaged the trials for each condition and participant separately. Since the contrast modulation was time-locked to the trial onset, the averaging of the time domains increased the signal-to-noise ratio of the steady-state EEG response.

To obtain the frequency spectrum of the averaged EEG we used the fast Fourier transform (FFT) after applying a Hanning window of 10% of the segment length. The frequency resolution of the spectrum was 0.1 Hz.

To define the region of interest, we averaged the amplitude of the frequency spectra across all conditions and all participants and found 14 electrodes with the maximal amplitude of the largest fundamentals. These electrodes were over the occipital areas (Fig. 3).

In order to determine the most prominent frequency components, we averaged separately for each series of experiment: the amplitude of the frequency spectra of all conditions; 14 occipital electrodes; and the results of all participants. Next, we computed a Z-score for each frequency bin by calculating the difference between the amplitude of the FFT value at the bin and the mean amplitude of five surrounding frequency bins on both sides (excluding one bin adjacent to the bin of interest). We then divided this difference by the standard deviation of the same surrounding bins^18^,^20^,^24^. We set a 99% threshold on the Z-values (Z = 2.33, p < 0.01, one-tailed: signal >noise) and detected the signals above the threshold. We excluded two participants in which neither fundamentals nor harmonics survived the thresholding. For further analyses, we selected the frequency components having Z-values higher than the threshold.

In the first series of the experiment, eleven frequency components survived the Z-score thresholding: two fundamentals (f_1_ = 7.50 Hz and f_2_ = 5.45 Hz), three harmonics (2f_1_ = 10.90 Hz and 2f_2_ = 15.00 Hz, 3f_2_ = 16.36 Hz) and six IMs (three summation frequencies: f_1_ + f_2_ = 12.9 Hz, f_1_ + 2f_2_ = 18.40 Hz, 2f_1_ + f_2_ = 20.45 Hz; three subtraction frequencies: 3f_2_-f_1_ = 8.86 Hz, 2f_1_-f_2_ = 9.55 Hz, 3f_1_-f_2_ = 17.04 Hz). In the second series, eleven frequency components also survived: two fundamentals (f_1_ = 4.00 Hz and f_2_ = 2.86 Hz), three harmonics (2f_1_ = 8.00 Hz, 2f_2_ = 5.71 Hz, 3f_1_ = 12 Hz) and six IMs (summation frequencies: f_1_ + f_2_ = 6.86 Hz, f_1_ + 2f_2_ = 9.71 Hz, 2f_1_ + f_2_ = 10.86 Hz, 2(f_1_ + f_2_) = 13.71 Hz, 3f_1+_f_2_ = 14.86 Hz, 4f_1_ + f_2_ = 18.86 Hz). Among these frequency components, we looked for those frequency types that were common for two series of the experiment. The common types were two fundamentals, two harmonics (2f_1_, 2f_2_) and three IM components (f_1_ + f_2_, f_1_ + 2f_2_, and 2f_1_ + f_2_).

For each condition and participant, we averaged the amplitude of 14 occipital electrodes and computed a SNR^19^ by dividing the amplitude of a FFT value by the mean amplitude of five surrounding frequency bins from both sides of this component (excluding one bin adjacent to the bin of interest). The usage of the SNR spectrum instead of the amplitude spectrum is a common practice in the SSVEP and frequency tagging research^14^,^18^,^19^,^20^,^24^,^25^. Since only small fraction of the noise is relevant with the frequency of interest^26^, the SNR spectrum provides much clearer brain responses (i.e., sharper spectral peaks) than the amplitude spectrum, especially for low frequencies. Another advantage of using the SNR in our study is as follows. The power of the EEG signal significantly decreases with frequency. The SNR computed relative to the adjacent frequency bins appears to be normalized for the background level of EEG power. This converted the data from two frequency sets to the same scale, allowing their merging. Therefore, after SNR calculation the SNR values for the frequency types, which were common for both series of the experiment, were used for statistical analyses.

## Additional Information

**How to cite this article**: Nihan, A. *et al*. EEG frequency tagging dissociates between neural processing of motion synchrony and human quality of multiple point-light dancers. *Sci. Rep.* **7**, 44012; doi: 10.1038/srep44012 (2017).

**Publisher's note:** Springer Nature remains neutral with regard to jurisdictional claims in published maps and institutional affiliations.

## Supplementary Material

Supplementary Information

## Figures and Tables

**Table 1:** The results of the 2 × 2 ANOVA on SNR data for fundamentals, harmonics and IM components (N = 38).

	Motion Synchrony					Human Configuration					Motion Synchrony X Human Configuration				
	SS	MS	F	p	η^2^	SS	MS	F	p	η^2^	SS	MS	F	p	η^2^
f1	66.53	66.53	1.97	0.16	0.05	1.90	1.90	0.04	0.82	0.00	35.30	35.30	1.40	0.24	0.04
f2	4.40	4.40	0.41	0.52	0.01	0.80	0.80	0.03	0.85	0.00	62.48	62.48	6.43	0.01*	0.15
2f1	18.37	18.37	0.96	0.33	0.00	2.04	2.04	0.10	0.74	0.03	12.64	12.64	0.81	0.37	0.02
2f2	14.88	14.88	1.96	0.16	0.09	18.74	18.74	3.77	0.05	0.05	11.55	11.55	0.60	0.44	0.02
f1 + f2	314.9	314.9	16.78	0.00**	0.31	126.8	126.8	2.83	0.10	0.07	26.72	26.72	0.95	0.33	0.025
2f1 + f2	22.20	22.20	1.92	0.17	0.04	32.74	32.74	4.39	0.04*	0.10	0.93	0.93	0.07	0.78	0.002
f1 + 2f2	0.42	0.42	0.08	0.76	0.002	16.97	16.97	3.11	0.08	0.07	0.46	0.46	0.06	0.80	0.001
