# Supplementary material for: EEG frequency tagging dissociates between neural processing of motion synchrony and human quality of multiple point-light dancers
Source: Sci Rep. 2017 Mar 8;7:44012. doi: 10.1038/srep44012 (PMC5341056; doi:10.1038/srep44012)
Supplement: Supplementary Information [file srep44012-s1.pdf]

Supplementary Information

EEG frequency tagging dissociates between neural processing of motion synchrony and human quality of multiple point-light dancers

Authors: Nihan Alp[1*], Andrey R. Nikolaev[2], Johan Wagemans[1], and Naoki Kogo[1]

Affiliations:

[1]Laboratory of Experimental Psychology, Brain & Cognition Research Unit, University of Leuven (KU Leuven), Leuven, Belgium

[2]Laboratory for Perceptual Dynamics, Brain & Cognition Research Unit, University of Leuven (KU Leuven), Leuven, Belgium

*nihan.alp@kuleuven.be

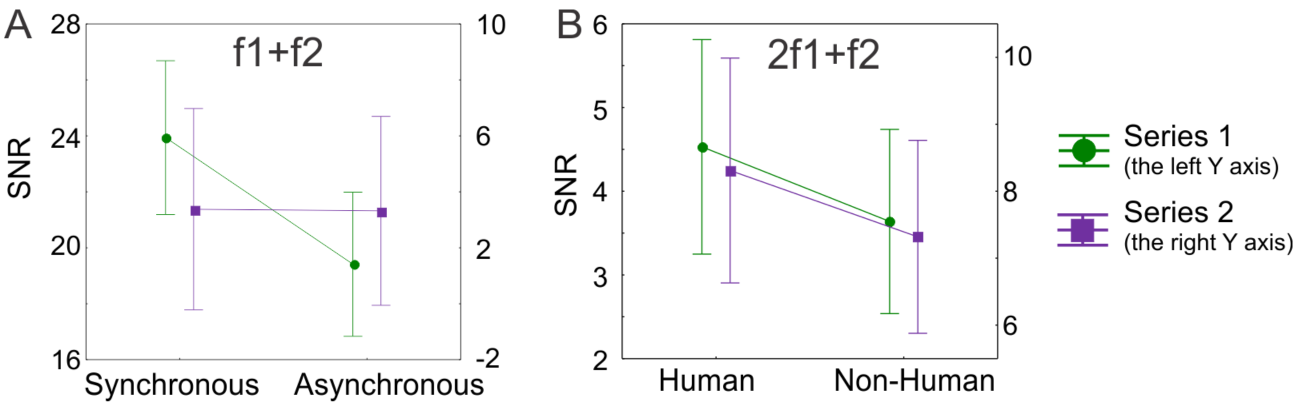

Fig. S1. Effect of motion synchrony and human configuration of a group of PLDs on SNR at the intermodulation components in two series of the experiment. (A) the effect of motion synchrony on SNR at the second-order IM component (f_1+f_2) per series: the SNR is significantly higher for synchronous than asynchronous motions in series 1 while it is constant in series 2. (B) the effect of human configuration on SNR at the third-order IM component ($2f_1+f_2$) per series: the SNR is higher for a group of human than for a group of non-human configurations.

Table S1A. Series 1 (the frequency set 1, N=24). The results of the 2 x 2 ANOVA on SNR data for fundamentals, harmonics and IM components.

	Motion Synchrony					Human Configuration					Motion Synchrony X Human Configuration				
Series1	SS	MS	F	p	η^2	SS	MS	F	p	η^2	SS	MS	F	p	η^2
f1	2.62	2.62	0.06	0.80	0.0	17.72	17.72	0.31	0.57	0.01	73.63	73.63	2.18	0.15	0.09
f2	14.33	14.33	1.15	0.29	0.05	6.60	6.60	0.22	0.63	0.01	58.54	58.54	4.42	0.04*	0.16
2f1	6.29	6.29	0.24	0.62	0.01	23.44	23.44	0.93	0.34	0.04	37.35	37.35	1.76	0.19	0.07
2f2	6.52	6.52	1.08	0.30	0.05	21.22	21.22	1.86	0.18	0.08	18.36	18.36	0.61	0.44	0.03
f1+f2	491.31	491.31	23.68	0.000065***	0.51	175.22	175.22	2.58	0.12	0.10	38.60	38.60	0.91	0.34	0.04
2f1+f2	4.52	4.52	0.77	0.38	0.03	19.09	19.09	2.29	0.14	0.09	14.18	14.18	1.31	0.26	0.05
f1+2f2	0.31	0.31	0.3	0.055	0.00	19.64	19.64	353	0.07	0.13	3.41	3.41	0.47	0.49	0.02

Table S1B. Series 2 (the frequency set 2, N=14). The results of the 2 x 2 ANOVA on SNR data for fundamentals, harmonics and IM components.

	Motion Synchrony					Human Configuration					Motion Synchrony X Human Configuration				
Series2	SS	MS	F	p	η^2	SS	MS	F	p	η^2	SS	MS	F	p	η^2
f1	128.14	128.14	6.37	0.02*	0.33	60.54	60.54	10.73	0.006**	0.45	2.09	2.09	0.24	0.62	0.02
f2	2.24	2.24	0.31	0.58	0.02	23.40	23.40	2.41	0.14	0.16	9.031	9.031	2.34	0.14	0.15
2f1	0.86	0.86	0.09	0.76	0.01	0.52	0.52	0.05	0.80	0.0	4.59	4.59	1	0.33	0.07
2f2	14.35	14.35	4.30	0.05	0.25	0.10	0.10	0.10	0.75	0.01	0.0001	0.0001	0.00013	0.99	0.00
f1+f2	0.04	0.04	0.01	0.90	0.00	1.49	1.49	0.40	0.53	0.03	0.14	0.14	0.03	0.85	0.00
2f1+f2	24.77	24.77	1.12	0.30	0.08	13.73	13.73	2.12	0.16	0.14	42.53	42.53	3.74	0.07	0.22
f1+2f2	0.11	0.11	0.02	0.86	0.00	0.97	0.97	0.17	0.67	0.01	12.52	12.52	1.80	0.20	0.12

Table S2. The control analysis with equalized series sizes (14 participants were randomly selected from series 1 and all 14 participants were included from series 2 (N=28)). The results of the 2 x 2 ANOVA on SNR data for fundamentals, harmonics and IM components.

	Motion Synchrony					Human Configuration					Motion Synchrony X Human Configuration				
	SS	MS	F	p	η^2	SS	MS	F	p	η^2	SS	MS	F	p	η^2
f1	20.68	20.68	0.48	0.49	0.02	54.70	54.70	1.53	0.23	0.05	4.43	4.43	0.18	0.68	0.01
f2	1.18	1.18	0.10	0.76	0.00	7.14	7.14	0.31	0.58	0.01	21.37	21.37	1.85	0.18	0.06
2f1	1.25	1.25	0.09	0.77	0.00	15.38	15.38	1.05	0.32	0.04	14.15	14.15	1.18	0.29	0.04
2f2	28.37	28.37	6.83	0.01	0.20	9.80	9.80	1.29	0.27	0.05	9.61	9.61	1.23	0.28	0.04
f1+f2	196.56	196.56	11.12	0.00**	0.29	132.20	132.20	11.12	0.09	0.29	34.32	34.32	2.05	0.16	0.07
2f1+f2	29.95	29.95	2.16	0.15	0.07	30.64	30.64	4.52	0.04*	0.14	14.94	14.94	1.01	0.32	0.03
f1+2f2	4.27	4.27	0.75	0.39	0.02	24.46	24.46	3.93	0.057	0.12	1.01	1.01	0.10	0.74	0.003
